# Complete Genome of *Bacillus megaterium* Siphophage Slash

**DOI:** 10.1128/genomeA.00862-13

**Published:** 2013-12-12

**Authors:** Andrew J. DeCrescenzo, Matthew A. Ritter, Karthik R. Chamakura, Gabriel F. Kuty Everett

**Affiliations:** Center for Phage Technology, Texas A&M University, College Station, Texas, USA

## Abstract

The complete annotated genome sequence of *Bacillus megaterium* bacteriophage Slash is described here. Several key features related to morphogenesis, replication/recombination, DNA metabolism, and lysis are described. Slash also encodes a homolog of SleB, a germination-specific cell wall amidase.

## GENOME ANNOUNCEMENT

*Bacillus megaterium* is a ubiquitous, spore-forming, Gram-positive bacterium (1). *B. megaterium* has been shown to be of great interest to the pharmaceutical industry, and it is used in production of penicillin, antifungals, and antivirals. The bacterium has important use as a model organism, as it is exploited in studies of membrane formation, the sporulation process, and protein localization (2, 3).

Bacteriophage Slash was obtained from a soil sample collected in College Station, TX. Phage DNA was sequenced using 454 pyrosequencing at the Emory GRA Genome Center (Emory University, Atlanta, GA). Trimmed FLX Titanium reads were assembled to a single contig at 111-fold coverage using the Newbler assembler version 2.5.3 (454 Life Sciences), with the default settings. PCR confirmed the completed contig. The genes were initially predicted using GeneMarkS (4), and gene predictions were corrected using software tools available on the Center for Phage Technology (CPT) portal (https://cpt.tamu.edu/cpt-software/portal/). Transmission electron microscopy was performed at the Microscopy and Imaging Center at Texas A&M University.

Phage Slash is a siphophage containing a 79,815-bp double-stranded DNA (dsDNA) genome with a G+C content of 35.6% and a coding density of 90.1%. Thirty-two genes were assigned functions by BLASTp and InterPro analyses (5, 6). In addition, 16 hypothetical conserved genes and 64 hypothetical novel genes were annotated.

Several morphogenesis genes were identified, encoding tape measure protein, head-to-tail joining protein, tailspike protein, and the terminase large subunit. Although the large terminase lacks homology to the terminases of known packaging strategies, an analysis of the raw sequencing data using the Pause method (https://cpt.tamu.edu/cpt-software/releases/pause/) revealed that Slash has a direct terminal repeat of 567 bp. The genes encoding DNA metabolism enzymes, including recombinase, DnaB-like helicase, DNA polymerase III (epsilon subunit), DNA primase, Holliday junction resolvase protein, and a RecB-like exonuclease, were identified. Slash also carries DNA biosynthesis genes (encoding dUTPase, thymidylate synthase, guanylate kinase, and the alpha and beta subunits of ribonucleotide reductase). Four putative sigma/transcription factors were also identified. Four homing endonucleases with unknown sequence specificities were identified, including two with an AP2 domain. The AP2 domain is a DNA binding domain that was originally identified in plant transcription factors (7). Recent structural analysis suggests that the plant AP2 domain is likely an evolutionary ancestor of homing endonucleases (8). A lysis cassette encoding a class II holin (two transmembrane domains in an N-in C-in topology) and an endolysin (l-alanyl-d-glutamate peptidase) was identified. Another muralytic enzyme (SleB-like, *N*-acetylmuramyl-l-alanine amidase) is encoded by a gene outside of the lysis cassette.

Gene 103 encodes a LysM/SCP domain-containing protein. The LysM domain is a peptidoglycan binding domain that is found in a variety of endolysins and bacterial pathogenesis factors (9). Proteins that contain the SCP domain are members of a larger superfamily of cysteine-rich secretory, antigen 5, and pathogenesis-related (CAP) proteins (10, 11). In addition to this potential lysogenic conversion gene and the SleB homolog, the presence of a Rha prophage regulator and an integrase/recombinase suggests that Slash may be a temperate phage.

### Nucleotide sequence accession number.

The genome sequence of phage Slash was contributed to GenBank as accession no. KF669661.
